# Bridging the Gap between Alzheimer’s Disease and Alzheimer’s-like Diseases in Animals

**DOI:** 10.3390/ijms20071664

**Published:** 2019-04-03

**Authors:** Anita Gołaszewska, Wojciech Bik, Tomasz Motyl, Arkadiusz Orzechowski

**Affiliations:** 1 Department of Physiological Sciences, Faculty of Veterinary Medicine, Warsaw University of Life Sciences—SGGW, Nowoursynowska 159, 02-776 Warsaw, Poland; an_ita@wp.pl (A.G.); mail@tomaszmotyl.pl (T.M.); 2 Department of Neuroendocrinology, Centre of Postgraduate Medical Education, Marymoncka 99/103, 01-813 Warsaw, Poland; wicedyrdn@cmkp.edu.pl

**Keywords:** longevity, Alzheimer’s pathology, amyloidosis, animal models, aging, brain, senile plaques, neurofibrillary tangles, cognition

## Abstract

The average life span steadily grows in humans and in animals kept as pets or left in sanctuaries making the issue of elderly-associated cognitive impairment a hot-spot for scientists. Alzheimer’s disease (AD) is the most prevalent cause of progressive mental deterioration in aging humans, and there is a growing body of evidence that similar disorders (Alzheimer’s-like diseases, ALD) are observed in animals, more than ever found in senescent individuals. This review reveals up to date knowledge in pathogenesis, hallmarks, diagnostic approaches and modalities in AD faced up with ALD related to different animal species. If found at necropsy, there are striking similarities between senile plaques (SP) and neurofibrillary tangles (NFT) in human and animal brains. Also, the set of clinical symptoms in ALD resembles that observed in AD. At molecular and microscopic levels, the human and animal brain histopathology in AD and ALD shows a great resemblance. AD is fatal, and the etiology is still unknown, although the myriad of efforts and techniques were employed in order to decipher the molecular mechanisms of disease onset and its progression. Nowadays, according to an increasing number of cases reported in animals, apparently, biochemistry of AD and ALD has a lot in common. Described observations point to the importance of extensive in vivo models and extensive pre-clinical studies on aging animals as a suitable model for AD disease.

## 1. Introduction

Aging is a process of gradual, progressive impairment of normal tissue function and whole-body homeostasis [1]. Nowadays, the progression of new technologies, health care and nutrition results in a longer life span of humans, as well as animals, especially accompanying and breeding ones. The recent study conducted on animals by Herculano-Houzel [2] suggests that longevity scales directly with the number of cortical neurons. According to the World Health Organization, the proportion of the world human population over 60 years old will reach up to 22% by 2050, whereas now it is about 12% [3]. In parallel with humans, the life expectancy of animals is also increasing. According to the American Veterinary Medical Foundation, the number of pet cats and dogs over 6 and 10 years old has increased by 6% and 15% respectively in the last 20 years [4]. Alzheimer’s disease (AD) is the most common neuroendocrine disorder in aging human beings. It is estimated that AD affects about 50 million humans worldwide [5] and is an absolute leader when it comes to causes of dementia among people over 60 years old. Clinical manifestations of AD result from injuries in the brain regions which control cognitive processes, especially in the hippocampus.

## 2. Aging and Pathogenesis of Alzheimer’s Disease (AD)

Fundamental roles in AD pathology are played by two hallmarks of this disease: amyloid beta (Aβ), a product of harmful amyloid precursor protein (APP) processing, and neurofibrillary tangles (NFT). AD is featured by extracellular accumulation of so-called senile plaques made of Aβ, and intracellular NFT aggregates made of hyperphosphorylated tau protein. These hallmarks are followed by an inflammatory reaction associated with local oxidative stress only. However, AD is not associated just with molecular and morphological changes. From the clinical point of view, it leads to changes in behavioral and cognitive functions. Even though it cannot be called Alzheimer’s, many animals seem to share forms of cognitive dysfunction very similar to AD which we called ALD. Regarding the fact that vast majority of pet owners (63.2%), considers their pets to be a family member [4], it should be vital for us to study ALD in context of animal dementia.

*Amyloid*—a word meaning “starch-like”, was firstly introduced by Rudolf Virchow in 1854 [6]. The pathologist used this term to denote some macroscopic abnormalities that he observed in human livers. He noticed that the abnormalities seemed to resemble some chemical properties of starch as they exhibited a positive iodine staining reaction. Amyloids were mistakenly believed to be related to starch for many years, but even after proving this wrong, the term was kept. Amyloid fibril formation is related to numerous incurable diseases, such as familial Mediterranean fever, myeloma, Creutzfeldt-Jakob disease or Down syndrome [7], but probably the one that is the best known is Alzheimer’s disease or Alzheimer’s-like diseases in animals, where the crucial role is played by the amyloid beta peptide.

**Figure 1:**
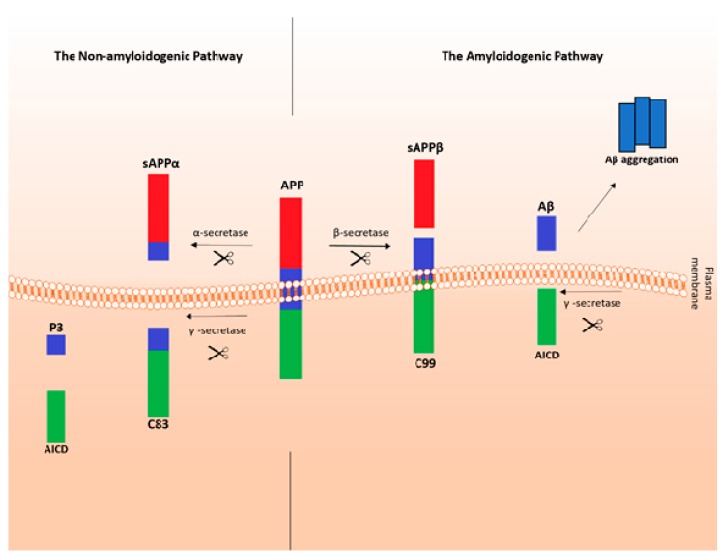
Amyloid precursor protein (APP) processing pathways. APP, a transmembrane protein may be processed in two pathways: the amyloidogenic pathway (β-secretase pathway) and the non-amyloidogenic pathway (α-secretase pathway). In the non-amyloidogenic pathway, α-secretase cleaves APP in a way that prevents the formation of amyloid-β (Aβ). It releases the APP C-terminal fragment 83 (C83) and soluble amyloid precursor protein α (sAPPα). C83 is later cleaved by γ-secretase to release the APP intracellular domain (AICD) and P3 fragment. In the amyloidogenic pathway, in turn, β-secretase cleaves APP to release sAPPβ (soluble amyloid precursor protein β), and the APP C-terminal fragment 99 (C99). C99 is later cleaved by γ-secretase, resulting in the release of Aβ and AICD.

Amyloid beta is produced by endoproteolysis of the parental amyloid precursor protein (APP), encoded by the *APP* gene. The cleavage and processing of APP are carried out by the group of enzymes or enzyme complexes, named α-, β-, and γ-secretase, and depending on enzymes that take part in the process, it can be divided into two pathways: a non-amyloidogenic pathway and amyloidogenic pathway (Figure 1).

In a prevalent, non-amyloidogenic pathway, APP is firstly cleaved by the α-secretase at a position 83 amino acids from the carboxy (C) terminus. So far, three enzymes with α-secretase activity have been identified, all belonging to the ADAM (a disintegrin- and metalloproteinase-family enzymes) family: ADAM9, ADAM10 and ADAM17 [8]. The cleavage results in producing a large amino (N)-terminal ectodomain called soluble amyloid precursor protein α (sAPPα), which is secreted into the extracellular compartment [9]. Studies suggest that sAPPα may have neuroprotective properties [10], and it is believed to be one of many potential treatments for AD [11]. The remaining, 83-amino acid C-terminal fragment (C83, CTF83) retains in the membrane and is later cleaved by a γ-secretase, producing a short fragment named P3 and a fragment called the APP intracellular domain (AICD). The reason why this pathway is not amyloidogenic is because of the cleavage by an α-secretase that occurs within the Aβ region, which precludes the formation of Aβ.

The amyloidogenic pathway is an alternative APP cleavage pathway, which leads to Aβ formation (Figure 1). Except for cats and rodents, the amino acid sequence of Aβ is well conserved among mammals [12]. The first stage of Aβ formation is carried out by an enzyme called β-secretase, which cleaves APP at a position located 99 amino acids from the C terminus. The initial proteolysis results in the release of sAPPβ (soluble amyloid precursor protein β) into the extracellular space. It also leaves a 99-amino acid C terminal fragment (C99, APP-CT-99) in the cell membrane. Subsequent cleavage of this remaining fragment by a γ-secretase (between residues 38 and 43) releases Aβ (into the extracellular space), and the AICD.

Cleavage by γ-secretase is somewhat imprecise resulting in heterogeneity of the cleaved peptide population. Numerous different Aβ types exist, but those which are 40 residues in length (Aβ40, Aβ1-40) are the most frequent (~80–90%), followed by the ones that are 42 residues long (Aβ42, Aβ1-42) (~5–10%) [13]. The longer forms of Aβ have stronger hydrophobic and fibrillogenic properties, and they are found to be the main Aβ forms deposited in the brain [14]. Interestingly, it should be pointed out that Aβ at the nanomolar concentrations (0.1-1 nM) inhibits the oxidation of blood plasma and cerebrospinal fluid (CSF) lipoproteins [15]. Moreover, it may have a role in the neuronal development [16] and the regulation of neurotransmission [17]. Aβ can also accumulate along cerebral blood vessels, causing a pathology known as cerebral amyloid angiopathy that is very often found in the course of AD.

Assemblies of Aβ can be divided into three groups based on their molecular weight, microscopic dimensions, and length: Aβ-derived ligands, protofibrils, and short oligomers [16]. Protofibrils and oligomers are believed to display the strongest effects in neurons, leading to neurotoxicity and synaptic disruption [18]. However, the soluble oligomers are thought to be more toxic than insoluble aggregates of protofibrils, and their levels correlate with Alzheimer’s disease severity better than the number of senile plaques [19]. Accumulation of Aβ leads to the formation of so-called senile plaques (SP). Although SP are thought to be one of the hallmarks of AD, they are frequently found in brains of cognitively normal, elderly individuals. Moreover, such age-related SP have been found in the brains of aged bears, carnivores, birds, cheetahs, domestic cats and many primates such as apes, and new and old world monkeys [20].

Neurofibrillary tangles (NFT) are one of the tauopathies. They are deposits of misfolded and hyperphosphorylated tau protein fibrils in the neuronal cytoplasm. Under normal conditions, tau stabilizes thick filaments as a microtubule-associated protein (MAP). Once it is phosphorylated, it falls off the microtubules, causing both loss of structural stability of the neurons, and disruption of the cell-trafficking. It should be highlighted that after the death of neurons containing NFTs, the remains of tangles reside in the brain as extraneuronal “ghost tangles”. Tau fibrils exhibit very strong neurotoxic and synaptotoxic properties [21]. Their presence and progression of formation correlate with the severity of cognitive decline in Alzheimer’s disease. They are found most frequently in the hippocampus, entorhinal cortex and isocortex [22]. Typical neurofibrillary tangles have never been denoted in animals. However, other forms of tauopathies are frequently observed in several species, including primates, bears, cheetahs, domestic cats, dogs, sheep, and goats [12].

The two hallmarks, NFTs and SP, play crucial roles in the pathogenesis of AD and most likely ALD. Their appearance is believed to lead to neuroinflammation that, in turn, results in increased production of reactive oxygen species (ROS) and reactive nitrogen species (RNS), such as superoxide anion radical (O_2_●–), hydrogen peroxide (H_2_O_2_), hydroxyl radical (HO●–), nitric oxide (NO●), and peroxynitrite (ONOO–). This condition is called oxidative stress and may lead to the damage of vital cellular elements, such as nucleic acids, lipids and proteins [23]. However, not only NFTs, Aβ accumulation and inflammation are believed to trigger oxidative stress, but it can also be caused by mitochondrial dysfunction and, according to recent data, metal accumulation [24]. Until now, it is still not known where and how oxidative stress originates [25].

## 3. Inflammatory and Immune Responses to AD

Oxidative stress can also be a trigger itself for the cell mediators of neuroinflammation. Microglial cells are derived from the mesoderm and constitute up to 12% of the total cell population in the brain [26]. They display characteristics of phagocytes, such as scavenger receptors and major histocompatibility complex II (MHC II) proteins. They also represent the first line of defense against pathogens and other types of brain tissue injuries. Under physiological conditions, microglia exhibit a “branched” morphology: large cell soma with thin, ramified processes. This state is usually called “resting microglia” or “ramified microglia”. Under pathological situations, such as injuries, oxidative stress, and other neurodegenerative conditions, microglia cells become activated. Their shape changes and becomes amoeboid, and they exhibit altered, upregulated expression of the cell surface markers, such as MHC II antigens. Most importantly, they start producing a large variety of growth factors, cytokines, chemokines, nitric oxide and reactive oxygen species [27]. These substances have cytoprotective properties and induce repairing processes in the brain tissues. For example, they are able to reduce Aβ accumulation by receptor-mediated phagocytosis and proteolytic enzyme degradation [28,29]. This kind of activation is called M2 activation, and it can possibly ameliorate the progression of AD [30]. However, sustained activation of microglia leads to a chronic inflammatory response and increased production of pro-inflammatory cytokines [31], which are synapto- and neurotoxic, and aggravate cognitive decline [32]. Moreover, activated microglia induce T lymphocytes to produce cytokines that are reactive towards myelin sheath’s proteins [33]. This kind of microglia activation is called M1 activation. Except for pathological conditions, it is also aging that causes changes in microglia, and this process is termed immunosenescence [34]. Activated microglia stimulate the activation and proliferation of astrocytes, cells that in physiological conditions maintain the neuronal environment and stabilize communication between the cells. There are elegant reviews [35,36] which address the dual role (neuroprotective and damaging) of the immune system. Additionally, there is a promising marker showing a rise in CSF of the immune response in AD [37]. Together with microglia, astrocytes support cognitive functions by controlling synaptic and structural plasticity [38]. In pathological conditions, these cells play an important role in Aβ degradation and protection of neurons against Aβ. They form a protective barrier along with microglial cells, surrounding amyloid plaques in their peripheries.

Both cells, microglia and astrocytes, express receptors that play important roles in the Aβ clearance. There is a plethora of such receptors known but the most important ones are believed to be toll-like receptors (TLRs) and scavenger receptors (SRs). Functionally, TLRs are involved in the uptake of Aβ and activation of microglia to produce proinflammatory mediators, following amyloid beta binding. SRs, in turn, exhibit only ability to induce Aβ uptake [39].

TLRs are mainly found in microglia, and are believed to be a front line in recognizing a wide spectrum of pathogens. They are also able to recognize Aβ peptides and oligomers. TLRs have been observed to exhibit both beneficial and noxious effects on AD-related pathologies [40]. After binding to TLR, Aβ activates the receptor and this can lead to neuroinflammation and microglial clearance of Aβ by phagocytosis. Interestingly, two forms of TLRs seem to play pivotal roles in AD: TLR2, and TLR4. Tahara et al. [41] showed that TLR4 knockout in transgenic AD mice increases numbers of Aβ deposits. Additionally, studies indicated that the expression of TLRs increases during pathological conditions, e.g., stress conditions or inflammation. This means that chronic inflammation may potentiate signal mediated by TLR resulting in aggravation of the AD pathology [42]. The expression of TLR4 is clearly increased in AD transgenic mice [43], and increased levels of TLR2 have been noticed in other AD animal models [44]. It was also proven that knockdown of TLR2 reduces expression of inflammatory molecules and lessens Aβ internalization by phagocytosis [30].

Scavenger receptors are named after their function. They are a broad family of membrane receptors, which can recognize and internalize many proteins including Aβ. Expression of SRs is upregulated in microglia in parallel with a number of Aβ deposits in Alzheimer’s disease brains [45,46]. Moreover, SRs have been reported to regulate the adhesion of mice microglial cells to Aβ, resulting in the production of reactive oxygen species, and Aβ uptake by microglia [47,48]. Deficiency of these receptors leads to impairment of Aβ clearance and accelerates ALD progression [49].

Glycogen synthase kinase-3 (GSK-3) is a highly conserved protein-serine/threonine kinase, which can be found in two forms: GSK-3α and GSK-3β. The second one, GSK-3β, is believed to a play a pivotal role in AD pathogenesis. It was first called tau protein kinase (TPK), since it was discovered in the microtubule fraction of the bovine brain, causing hyperphosphorylation of tau. In physiological conditions, it regulates many crucial cellular processes, controlling a myriad of signaling pathways. GSK-3β is mainly activated as a result of reduced cell survival signals for example growth factors. However, animal models of AD indicate that GSK-3β can also be activated by Aβ by preventing inhibitory phosphorylation of this enzyme [50,51]. Dysregulation of this enzyme occurs in the development of many diseases, including Alzheimer’s disease. It is involved in the formation of NFTs and Aβ-induced neuronal death [52], and the appearance of these two AD hallmarks leads to the axonal transport degradation, synapse loss and eventually neuronal death. Consistent with this, GSK-3β inhibition has been shown to reduce Aβ production in Alzheimer’s disease murine models, and to lessen Aβ-induced neurotoxicity in cultured neurons [53,54,55].

All of the lesions described above, and many more that are also believed to be AD features, lead to the mitochondrial decline in energy production, ROS generation and accumulation of its damaged products, inflammation, and loss of regenerative ability [56]. All of these mechanisms cause ongoing neurodegeneration leading to the brain atrophy. Importantly, all of the mentioned above neurodegenerative processes begin much earlier than the first clinical symptoms occur.

**Figure 2:**
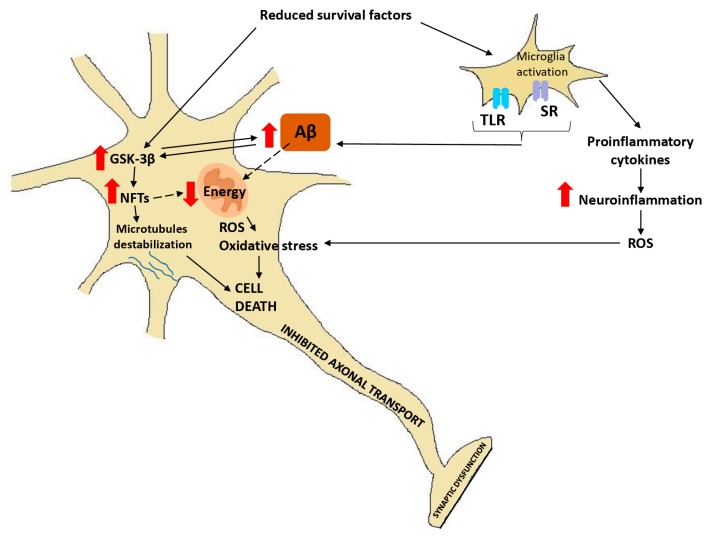
Summary of possible interactions of described responses in neuron dysfunction and death in AD.

Interestingly, in AD and in normal aging neuronal loss is not a prerequisite for functional deficits [57]. Alzheimer-like lesions and their consequences are often observed in the brains of cognitively normal individuals [58]. This is possible because of a process termed neuroplasticity. It involves modulation of structural and functional processes of neurons, e.g., synaptic remodeling, synaptogenesis, axonal sprouting, and dendritic remodeling. Summary of the possible interactions of responses in a neuron during AD is presented in Figure 2.

## 4. Animals and Neurodegenerative Diseases Including Alzheimer’s-like Disease

**Figure 3:**
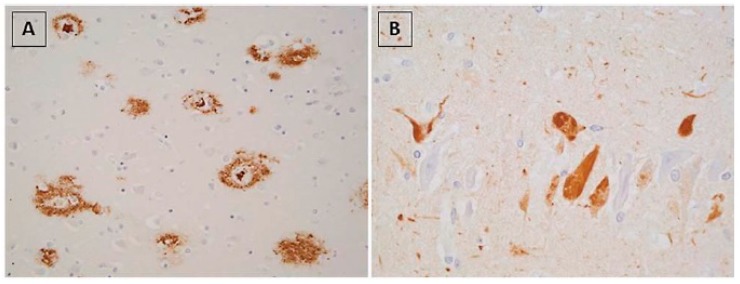
Alzheimer’s disease neuropathology in humans. (**A**) Senile plaques and globose diffuse deposits demonstrated with anti-Aβ antibody (M 0804, Dako). (**B**) Neurofibrillary tangles demonstrated by phosphorylated tau protein immunohistochemistry (PHF-tau; AT8, Thermo Scientific). Reprinted from *Journal of Alzheimer’s disease*, 53, Taipa R., Melo-Pires M., Sousa L., Sousa N.: Does the Interplay Between Aging and Neuroinflammation Modulate Alzheimer’s Disease Clinical Phenotypes? A Clinico-Pathological Perspective, 403–417, 2016, with permission from IOS Press [59].

The growing interest in neurodegenerative diseases in humans led to significant progress in the understanding of their patomechanisms. What is important, this interest has also caused intensified research on neurodegenerative diseases in animals, especially domestic ones. Interestingly, many neurodegenerative diseases in animals are very similar to their human counterparts in both, morphological (Figure 3.) and clinical ways.

Motor neuron diseases (MND) are degenerative disorders of the central nervous system (CNS) affecting motor neurons of the spinal cord, brain stem, and motor cortex. Animals with MND exhibit paralysis with progressive muscle atrophy, paresis, and impairment of controlling their limbs. Examples of the best-known MNDs among dogs are hereditary canine spinal muscular atrophy (HCSMA), Stockard’s paralysis, which has been reported in Great Danes, St. Bernard, and crossbreds [60], asymmetrical spinal muscular atrophy in German shepherd dogs [61], and many more. MNDs among cats are very uncommon but they have been reported in calves (e.g., Werdning–Hoffman disease), pigs and horses (equine motor neuron disease, EMND) [62,63].

Another group of neurodegenerative-derived disease includes degeneration of the autonomic neurons. These diseases have been reported not only in domestic animals but also in wild species. Clinical signs include dysphagia, tachycardia, cachexia, sweating, and peripheral vasodilatation. Examples of these diseases are equine grass sickness (equine dysautonomia), feline dysautonomia, and canine dysautonomia.

Furthermore, neurodegenerative processes may result in the neuronal degeneration of the brainstem and cerebrum, with mostly fatal consequences in humans and animals. Examples of such diseases in animals are: neuronal vacuolation in Rottweiler dogs [64], many forms of spongiform encephalopathies (bovine or feline spongiform encephalopathy, chronic waste disease, transmissible mink encephalopathy found in in cattle, cats, deers, and minks respectively), aging, dementia, Parkinson’s disease, and Alzheimer’s-like diseases [65].

With advances in modern veterinarian medicine, domestic animals often live long enough to develop cognitive dysfunction. Pets live in safe, controlled environments, have healthy diets and access to great medical care. They are no longer just animals as they have become members of families and humans’ best friends. Nowadays, people treat animals with bigger respect and concern than before. Although it is hard to name it Alzheimer’s disease exactly, animals often exhibit symptoms of this kind of disease (ALD) not only in behavior but also in histopathological view. As the detection of their age-related behavioral changes is rather easy for both: pet owners and veterinarians, they are easy to handle. Pet’s life span is moderate and they often share not only the environment but sometimes also the same kind of food with humans, they may provide a unique model for studies of human aging.

Domestic cats display several behavioral changes in their elderly years. The most common is spatial disorientation or confusion, for example getting trapped in the corners or forgetting the location of the litter box. It is also frequently observed that social relationships with their owners or other animals in the house are altered, e.g., cats become more aggressive or passive. Geriatric cats happen to change their daily schedule, including their wake-sleep pattern and their interest in food and they decrease grooming. They sometimes exhibit inappropriate vocalization, like crying loudly during the night [66,67,68].

**Figure 4:**
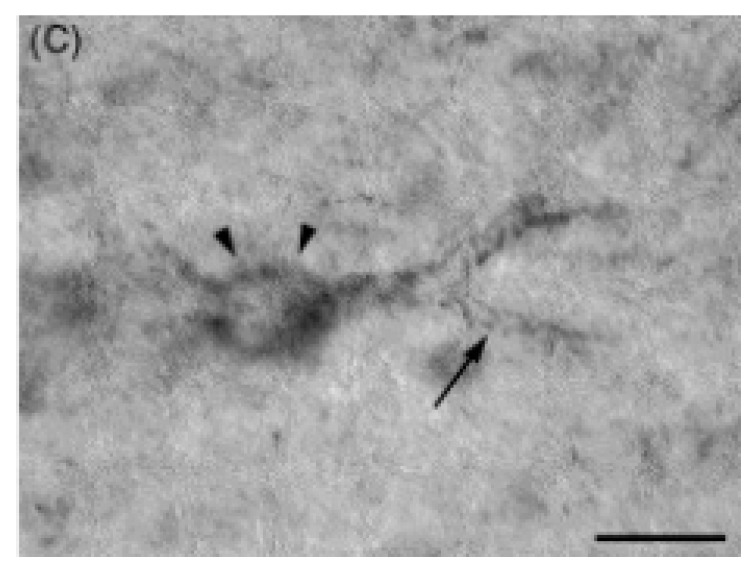
Aged cat’s brain. Individual neurons show an accumulation of Aβ1–42 (labeled with anti-Aβ1–42), both in apical dendrites (arrows) and within the somatodendritic compartment (arrowhead). Bar = 20 μm. Reprinted from *Neurobiology of Aging*, 26, Head E., Moffat K., Das P., Sarsoza F., Poon W.W., Landsberg G., Cotman C.W., Murphy M.P. β-Amyloid deposition and tau phosphorylation in clinically characterized aged cats., 749–763, 2005, with permission from Elsevier [70].

**Figure 5:**
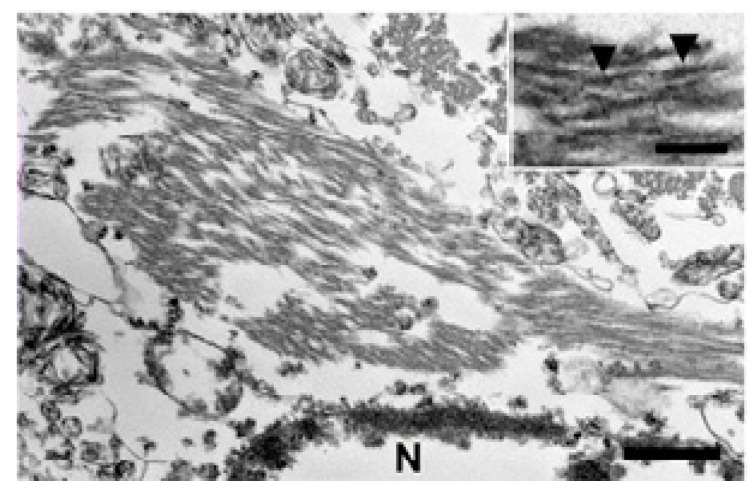
Neurofibrillary tangles in the cat hippocampus, transmission electron microscopy. Clusters of filaments can be found in the perikaryon in two kinds of forms: straight and paired twisted. In the case of the paired twisted form, the lengths between arrowheads were 80–100 nm. Bars = 500 nm and 100 nm (inset). N stands for Nucleus. Reprinted from *Acta Neuropathologica Communications*, 3, Chambers J.K., Tokuda T., Uchida K., Ishii R., Tatebe H., Takahashi E., Tomiyama T., Une Y., Nakayama H. The domestic cat as a natural animal model of Alzheimer’s disease, 78, 2015, with permission from BMC [71].

Several studies have identified senile plaques in the cats’ brains but only in those aged 10 and more [69,70]. However, Chambers [71] proved that Aβ can also aggregate as oligomers in younger cats, aged eight. Curiously, there were cases found with Aβ deposits that were not associated with tau immunoreactivity, but no cases were found with NFT in the absence of Aβ deposits. Additionally, in cat ALD, Aβ aggregates differently in the cerebral cortex and hippocampus, which may be due to different neuronal cell types in both regions, or/and environments surrounding neurons in these regions (Figure 4 and Figure 5).

The association between amyloid beta depositions in the brain and cognitive dysfunction in cats remains clarified, as it was proven multiple times, that the brains from aged cats, who exhibited altered behavior, were found to contain diffuse senile plaques [70,71]. Interestingly, SPs of cats seem to be more diffuse than those that can be found in dogs with ALD [72,73].

Since domestic cats can spontaneously develop Aβ deposition, neurofibrillary tangles formation, neuronal loss and neuronal degeneration (in contrast to other animals) during their short life-span, they could serve as a valuable natural model of human Alzheimer’s disease.

Dogs display patterns of cognitive impairment that are similar and comparable to humans [74]. A specific age-related syndrome known as canine cognitive dysfunction (CCD) exists in elderly dogs and is proven to share some features (both clinical and neuropathological) with early stages of Alzheimer’s disease [75].

Behavioral changes can be divided into four general categories, which are: loss of cognition and recognition, loss of house training, disorientation, and changes in their sleep-wake cycle. There is a scale termed CCDR (canine cognitive dysfunction rating), which allows assessing the severity of the cognitive dysfunction scale [76]. Affected dogs exhibit a change in behavior and daily routines, e.g., they do not recognize family members, forget former house-training, get lost in their houses, get stuck in the corners, and act peculiarly by whining, scratching the floor without reason, and barking a lot.

**Figure 6:**
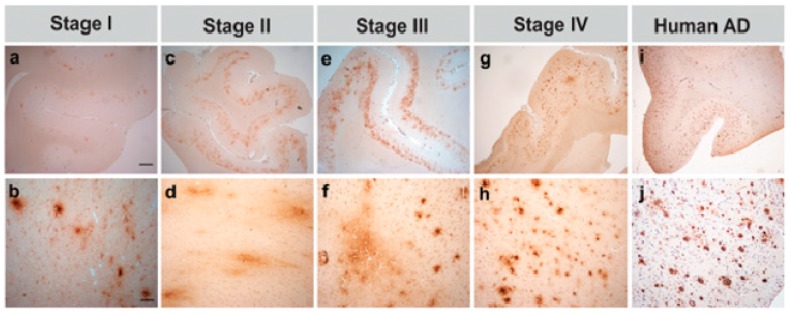
Photomicrographs exemplifying the four stages of amyloid beta (Aβ1-16) deposition in aged canines’ prefrontal cortex. (**a**,**b**) Stage I—very few small plaques with poorly defined borders. (**c**,**d**) Stage II—abundant, cloud-like plaques in the deep cortical layers. (**e**,**f**) Stage III—abundant, cloud-like plaques in the deep cortical layers and small, distinct plaques in facile layers. (**g**,**h**) Stage IV—meager, condensed plaques found in all cortical layers, except layer I. (**i**,**j**) Immunoreactivity of amyloid plaques in human’s frontal cortex (patient with Alzheimer’s disease). Bars = 1000 µm in (**a**,**c**,**e**,**g**,**i**), and 100 µm in (**b**,**d**,**f**,**h**,**j**) [75]. Reprinted from *Journal of Alzheimer’s disease*, 52, Schütt T., Helboe L., Pedersen L.O., Waldemar G., Mette B., Pedersen J.T.: Dogs with Cognitive Dysfunction as a Spontaneous Model for Early Alzheimer’s Disease: A Translational Study of Neuropathological and Inflammatory Markers, 433*–*449, 2010, with permission from IOS Press.

Neuropathological changes, which occur in the aging dogs’ brains or ALD, include the cortical atrophy, dysfunction in the neurotransmitter systems, increased oxidative damage, extracellular deposition of diffuse Aβ (Figure 6), ventricular enlargement, neuronal loss and decreased neurogenesis [75,77,78,79,80]. Dogs also develop some kind of tau abnormalities, but not regular neurofibrillary tangles [81]. Depending on the stage of their development, there are three significant types of amyloid plaques found in dogs. A diffuse plaque, which is a non-β-sheet; a primitive plaque, which is a β-sheet but lacking a central core of amyloid, and a neuritic plaque, which is a β-sheet containing a central core of amyloid and vast, reactive astroglia. Similarly to humans, the different plaque subtypes have different locations in the dog brain areas. The most common form found in the dog brain affected by ALD seems to be Aβ1–42, which is a component of the diffuse plaques, whereas mature (primitive or neuritic) plaques are usually formed by the Aβ1–40 [82]. Aβ deposits in the dogs’ brains can be found in the cerebral cortex, hippocampus and meningeal vessels in contrast to cats, in which Aβ cannot be found in meningeal vessels. Aβ deposition begins in the prefrontal cortex of a dog, and later occurs in the temporal and occipital cortex, similar to those reported in humans [83]. Intriguingly, oligomers of Aβ can be detected in the cerebrospinal fluid of dogs, but are inversely related to the amount of total amyloid beta measured biochemically in the brain, which may suggest that Aβ oligomers are sequestered into plaques [79].

Because of their natural age-related deposition of Aβ in the brain, along with their simply assessable cognitive functions, dogs are very useful models for investigating Alzheimer’s disease and plan therapies that target the disease. Moreover, a study confirmed, that not all aged dogs develop Aβ deposits in the brain, which suggests that, as in humans, external and/or genetic factors may determine the possibility of individuals for developing Aβ pathology at a given age [84,85,86].

There is a bulk of evidence showing Aβ cerebral deposition found in domestic carnivores and wild omnivores, and only a few reports described domestic and wild large herbivores. However, several papers have been published regarding horses [87], sheep [88], cattle [89], elephants [90], and camels [91].

**Figure 7:**
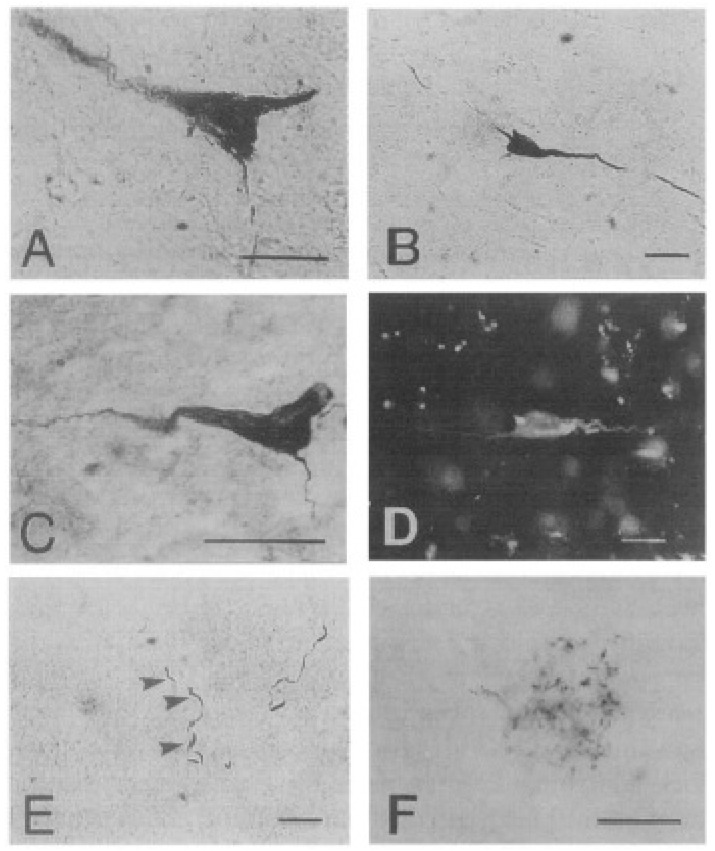
Neurofibrillary degeneration in the sheep cortex as viewed under the light microscope. Neurofibrillary tangle-like structures (**A***–***D**) in sheep brains, stained using: (A) Alz-50 antibody, (**B**) PHF-I antibody, (**C**) Gallyas’ silver stain, and (**D**) thioflavine S. Lesions resembling ‘neuropil threads’ (**E**) and neuritic plaques (**F**) could be visualized using PHF-I (shown here) as well as Alz-50. Bars in A = 20 µm, in F = 50 µm. Reprinted from *Neuroscience Letters*, 170, Nelson P.T., Greenberg S.G., Saper C.B. Neurofibrillary tangles in the cerebral cortex of sheep, 187–190, 1994, with permission from Elsevier [88].

Among the above species, the presence of neurofibrillary tangles has been reported only in sheep (Figure 7). Interestingly, some clear evidence of tau accumulation in horses was detected too, but the hippocampal neurons, which contained the accumulations, did not express hyperphosphorylated tau [87]. The conclusion was that non-phosphorylated tau may accumulate in some hippocampal neurons in a non-neurofibrillary tangle manner because of axonal transport deficiencies occurring in aging. The presence of NFTs has still not been detected in elephants, camels, and bovine brains.

**Figure 8:**
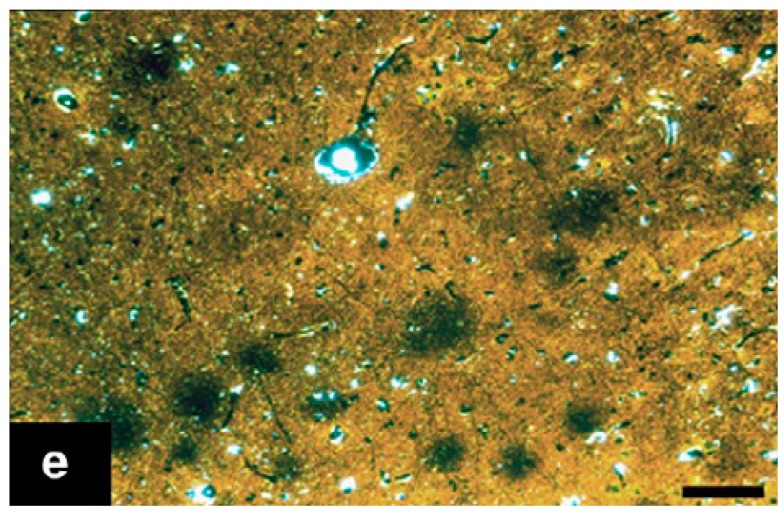
Diffuse plaques in the neocortex of a horse. Methenamine silver. Bar = 50 µm. Reprinted from *Journal of Comparative Pathology*, 142, Capucchio M.T., Marquez M., Pregel P., Foradada P., Bravo M., Mattutino G., Torre C., Schiffer D., Catalano D., Valenza F., Guarda F., Pumarola M. Parenchymal and Vascular Lesions in Ageing Equine Brains Histological and Immunohistochemical Studies, 61*–*73, 2010, with permission from Elsevier [87].

There have been no cases reported of cerebral Aβ deposition in sheep and elephants, unlike horses, in which brains diffuse Aβ plaques were found [87]. Interestingly, the plaques were characterized by the accumulation of Aβ42 and no Aβ40, similarly to dogs (Figure 8).

**Figure 9:**
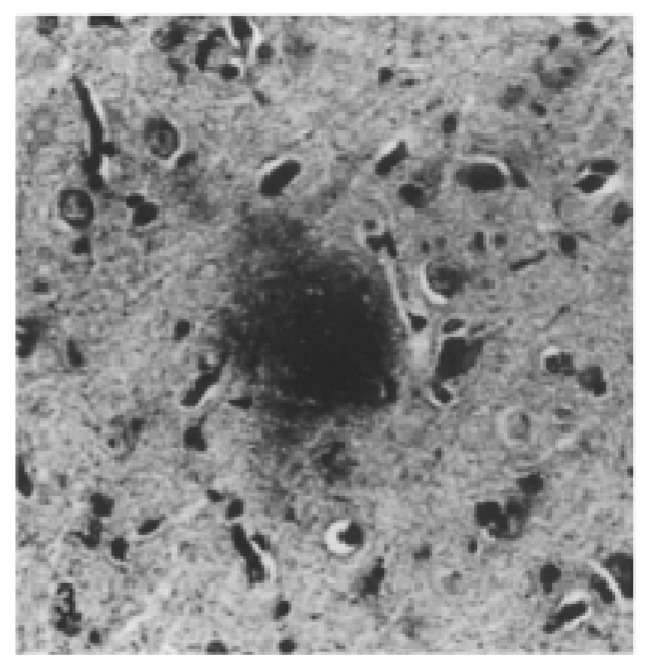
A diffuse plaque in the cerebral cortex of a camel. Periodic acid methenamine silver (PAM), ×225. Reprinted by permission from *Springer Nature*, Acta Neuropathologica, senile plaques in an aged two-humped (Bactrian) camel (*Camelus bactrianus*). Nakamura S., Nakayama H., Uetsuka K., Sasaki N., Uchida K., Goto N., 90, 415*–*418, 1995 [91].

Senile plaques detected in the brain of a 20-year-old camel affected by ALD were mostly of the diffuse type (Figure 9). They were mainly distributed throughout the cerebral cortex but absent in the hippocampus and the cerebellum. Interestingly, the camel case is the first herbivorous animal in which the presence of Aβ plaques has been detected on the basis of proper histopathological and immunohistochemical examinations.

In a study conducted by Costassa [89], an age-related progression of Aβ deposition in cattle brains was detected. Granular aggregates of Aβ peptides, without the presence of plaques, were observed.

According to Gunn-Moore et al. [92], sea mammals, such as dolphins and sea lions, might have AD-like neurodegeneration. It ranges from sea lions having CSF markers which can be also found in AD, to postmortem amyloid and tau pathology in the brains of dolphins.

Non-primate species have been particularly studied because of their genetic similarities with humans. Senile plaques and vascular amyloidosis have been described in baboons [93], squirrel monkeys [94], gorillas [95], chimpanzees, and macaques [96].

Aged chimpanzees displayed amyloid deposition in the cortical and meningeal vessel walls. Moreover, amyloid plaques in the brain parenchyma have also been reported [96]. Importantly, senile plaques in ALD were mainly of the diffuse type.

**Figure 10:**
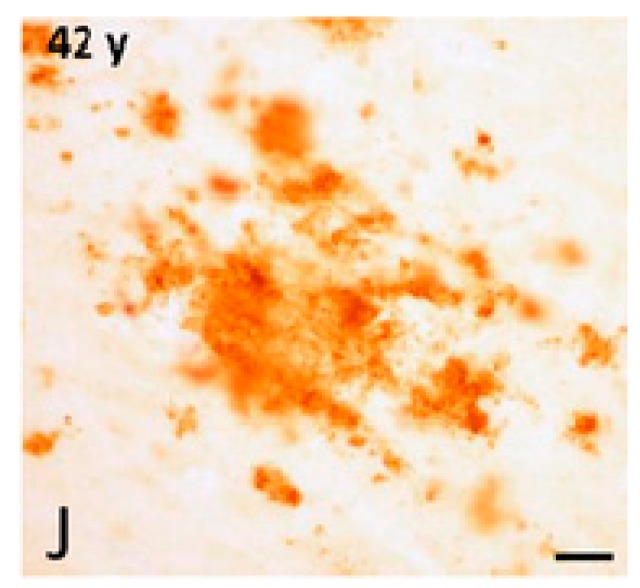
High-magnification photomicrographs showing a diffuse APP/Aβ-ir (immunoreactive) plaques in the frontal cortex of a 42-year-old female gorilla. Bar = 25 μm. Reprinted from *Neurobiology of Aging*, 39, Perez S.E., Sherwood C.C., Cranfield M.R., Erwin J.M., Mudakikwa A., Hof P.R., Mufson E.J. Early Alzheimer’s disease-type pathology in the frontal cortex of wild mountain gorillas (Gorilla beringei beringei), 195*–*201, 2016, with permission from Elsevier [97].

Deposits of Aβ were also observed in middle-aged Western Lowland gorillas (Figure 10). Plaques consisting of Aβ-42 were found in the cerebral neocortex and were of the diffuse type, showing filamentous and amorphous structures with an irregular margin [97]. It is possible that the diffuse plaques could evolve into senile plaques with time. Neurofibrillary tangles have not been reported yet.

**Figure 11:**
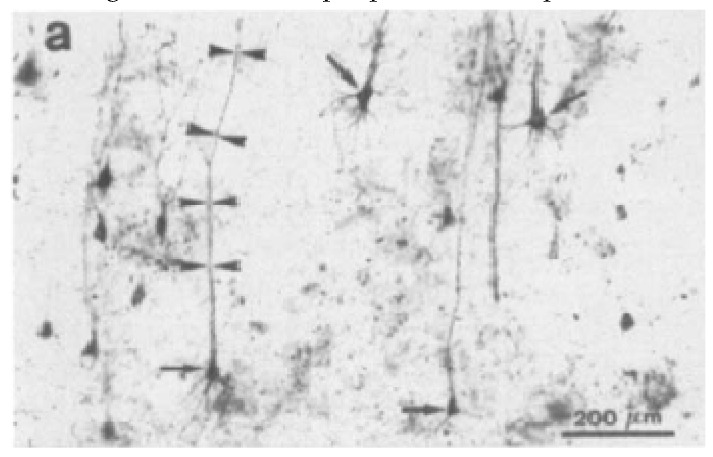
Cytoskeletal pathology in neurons of the baboon labeled by AT8-immunostaining. Deposition of abnormal tau in pyramidal neurons of the first Ammon’s hom sector (CA1). Abnormal tau is deposited in cell bodies (arrows) and apical dendrites (arrowheads) of CA1 pyramidal neurons (Bar = 200 µm; 30-year-old baboon). Reprinted by permission from Springer Nature, Advances in Experimental Medicine and Biology, Tau Pathology in Neurons and Glial Cells of Aged Baboons, Schulz C., Braak E., Tredici K., Hubbard G., Braak H., 2001 [98].

In baboons, brain plaques were also detected. Similarly, to gorillas and chimpanzees, plaques were of the diffuse, non-fibrillar type. Interestingly, in conjunction with the aforementioned species, a faint immunolabelling of hyperphosphorylated tau in the oldest baboons was found (Figure 11) [93]. This phenomenon is very similar to the situation observed in humans, where neurofibrillary tangles develop in the last stages of the diffuse plaque formation process.

In squirrel monkeys, not only diffuse plaques but also senile plaques have been reported to occur [94]. Resume of findings showing similarities between AD and ALD are listed in Table 1.

## 5. Identification of the Disease

Even though scientists keep trying to understand Alzheimer’s disease better and to find out a remedy for it, it still seems to be a long way to go until we get a wide, clear view of the disease, due to its complex and multifactorial nature. Because of this, nowadays a lot of effort is put into developing effective diagnostic of AD.

According to Blennow [99], biomarkers are „objective measures of a biological or pathogenic process, that can be used to evaluate disease risk or prognosis, to guide clinical diagnosis, or to monitor therapeutic interventions”. Since the cerebrospinal fluid is in constant, direct contact with the brain, it can indicate changes occurring in the brain tissue. Along with plasma, CSF is an excellent source of Alzheimer’s disease biomarkers.

Cerebrospinal fluid biomarkers can be divided into two groups: basic biomarkers and core biomarkers. Basic ones are used to determine and describe conditions, which may coexist with Alzheimer’s disease or pretend to actually be the disease. The latter biomarkers are used to identify the actual pathogenic processes in AD.

Among basic biomarkers, the following can be distinguished: cerebrospinal fluid cell count, CSF:serum albumin ratio and immunoglobulin G (IgG) or immunoglobulin M (IgM) index. The first one may indicate inflammation and is mainly used to exclude infections and infectious diseases. In AD, the number of cells in CSF remains unchanged, opposite to infections. Another biomarker, CSF:serum albumin ratio, allows showing whether the blood–brain barrier (BBB) functions properly or not. In pure, advanced AD the ratio is unchanged, while in mild cases with concomitant cerebrovascular pathology it may raise. The last basic biomarkers, IgG and IgM indexes, mirror intrathecal immunoglobulin production. In AD, they should stay unchanged, in contrast to infections, tumors, and inflammatory disorders.

Core biomarkers are the presence of Aβ1–42, p-tau181, p-tau231, and t-tau in CSF [100]. The first one indicates the presence of the amyloidogenic pathway of APP metabolism. The presence of phosphorylated tau protein (p-tau181, p-tau231) and t-tau (total tau) directly shows that the process of neuronal degradation has begun. All of the core biomarkers except Aβ1–42 are increased in Alzheimer’s disease. Concentrations of Aβ1–42 in CSF decrease in AD, since they become deposits known as “amyloid plaques” [101,102,103].

There are several publications trying to describe candidates of biomarkers of AD. Except for Aβ1–42 mentioned before, other products of Aβ amyloidogenic and non-amyloidogenic pathways are suggested, such as sAPPβ, sAPPγ and BACE1 [104,105,106]. Aggregation of not only Aβ oligomers but also other Aβ isoforms, such as Aβ1-13, Aβ1-14, Aβ1-15, Aβ1-16, Aβ1-17, and Aβ40 are also considered to play an important role in the detection of AD [102,106].

Not only levels of certain substances in CSF and blood plasma are studied in terms of AD biomarkers. Very promising methods of predicting possible Alzheimer’s disease are positron emission tomography (PET) and magnetic resonance imaging (MRI). Both of the methods have one great advantage in oppose to CSF and plasma assessment, they are not invasive for a patient since a lumbar puncture is not needed. It is very important in terms of working with elderly individuals and animals. Moreover, a special kind of PET, fluorodeoxyglucose positron emission tomography (FDG-PET), is believed to be the strongest predictor method for progression from mild cognitive impairment (MCI) to AD [107].

MRI has been used to measure the brain atrophy and its progression in patients with MCI, analyzing the volume of the hippocampus, entorhinal cortex and other regions (structural MRI). It can also be used to assess resting state functional connectivity (functional MRI, fMRI). PET, in turn, allows measuring the amyloid burden starting from very early stages of AD, thus helping with predicting Alzheimer’s disease development from MCI [108].

Growing literature data suggest that microRNAs (miRNAs) may serve as a novel AD marker. It is not a surprise since it was proven several times that miRNA profiles in CSF, plasma, and serum of healthy individuals differ from those suffering from AD [109,110,111]. Interestingly, different miRNAs are involved in particular stages of AD. Therefore, these molecules could be used not only as diagnostic biomarkers but also as a prognostic and therapeutic tool [111].

A lot of attention has been paid to nanotechnology as a new tool of not only diagnosis but also therapy for Alzheimer’s disease. Strategies involve using, for example, gold, liposomes, exosomes, magnetic nitrogen-doped graphene, iron oxide, mixtures of magnetic particles, and many more [112,113].

A promising diagnostic value has been noticed in measuring the blood pressure. However, it still requires to be investigated. A study on humans revealed that in individuals over 60 years old SBP (systolic blood pressure) was inversely associated with a prospective dementia diagnosis, whereas in the middle-aged subjects elevated systolic blood pressure and pulse pressure were associated with eventual AD in participants who reported using blood pressure lowering drugs [114].

## 6. Treatment Strategies

Treatment of Alzheimer’s disease seems to be one of the major challenges for the future of neurosciences. Currently available pharmacological therapies can only treat the symptoms of Alzheimer’s and have limited benefits. The main target of pursuing medications seems to be Aβ. In general, the efforts can be divided into three major groups: inhibiting amyloid aggregation, immunotherapy approaches (including passive and active immunization), and β- and γ-secretase inhibitions [115,116]. The three groups seem to be obvious choices of scientists’ interest. Importantly, all of them are very productive fields of research, even though they are incredibly complex and require the cooperation of scientists of many specializations. A very probable future for treatment of AD is a combinatorial approach, just like in cases of, e.g., hypertension, cancer, HIV and atherosclerosis. Such therapies in AD could be purportedly secretase inhibitor + immunotherapy; aggregation inhibitor + secretase inhibitor, and others [117].

Important roles in AD prevention and treatment are suggested to be played by proper diet, physical exercise, reinforcement training and playing interactive toys or food puzzles, which help to maintain brain health. According to Studzinski’s study conducted on aged dogs, a ketogenic diet may improve mitochondrial function and decrease APP levels in individuals with AD [118]. Physical exercise has been shown to increase growth factors, synaptic markers, neurothrophins and reduce neuroinflammation. However, it seems to be possible to be achieved under one important condition: an increased process of the hippocampal neurogenesis (AHN) is required to mediate the effect of exercise [119]. A similar study was conducted on humans as well. A trial named the Finnish Geriatric Intervention Study to Prevent Cognitive Impairment and Disability (FINGER) was carried between 2009 and 2011. Enrolled men were assigned to two groups: control, and treated one. Members of the latter group were directed to a regimen of cognitive training, exercise, and diet. After two years, it turned out that the treated group exhibited improvement in all of the investigated areas: complex memory, executive functioning, and processing speed [120].

An interesting solution for new AD therapeutics was recently proposed. Camargo et al. [121] suggested that peptides isolated from animal venoms could be new weapons in a battle against Alzheimer’s disease. Suggested venoms are from animals like for example *Daboia russelli russelli*, a subspecies of Russell’s viper found in eastern India [122], Buthus martensii Karsch scorpion [123], Eastern Green Mamba (*Dendroaspis angusticeps*) [124] and Taiwan banded krait (*Bungarus multicinctus*) [125]. Even though the knowledge about the use of those peptides in AD is insufficient, it seems plausible that this field will develop in the nearest future.

Many anti-amyloid beta immunotherapies targeting Aβ, as well soluble as insoluble, have been tested as ways of treating and preventing AD in humans and animals. A promising example can be immunotherapy based on a mixture of fibrillar Aβ components, which has shown very satisfying effectiveness along with absolute safety in domestic dogs with cognitive dysfunction syndrome. A study conducted by Bosch et al. [126] showed that such vaccine led to expeditious cognitive improvement in all of the treated animals. Immunized dogs tended to have smaller amyloid plaques in comparison with non-immunized dogs. Importantly, no side effects were observed in all individuals.

The bond between animal and human is nowadays extremely emotional, not to say, existential. There are many pieces of evidence supporting that such a relationship may have „healing power”. It was noted that elderly patients with dementia when eating in front of the aquarium, have an increased body mass compared to those eating isolated. Moreover, petting a dog or a cat causes increased secretion of “hormones of happiness”, such as oxytocin, serotonin, dopamine and prolactin, which is beneficial for nervous system and for fighting depression, which is often observed to accompany AD [127].

Accumulating evidence shows that AD is a metabolic disease and that scientists should focus more on metabolites that are affected by metabolic alterations in order to find a remedy for the disease. Importantly, many studies suggest that AD is strongly connected with Type 2 Diabetes Mellitus (T2D, T2DM). It was proven that the risk of developing Alzheimer’s disease is increased by 50–60% in case of suffering from T2D [128]. The link between the two diseases is so strong that scientists proposed a new term: Type 3 Diabetes (T3D). The term stands for Alzheimer’s disease induced by Type 2 Diabetes. Of note, disturbed glucose and insulin homeostasis have been observed in the brain in a course of AD.

There is also a growing body of evidence that metformin, a medication widely used in T2D treatment, has the potential of becoming a weapon against AD. Metformin influences insulin activity as it serves as insulin-sensitizing agent. Since a connection between AD and T2D is observed, drugs used in the latter disease are being deeply investigated towards therapeutic potentials in the neurodegenerative diseases. An important trait of metformin in terms of AD treatment seems to be that it has anti-inflammatory properties. Moreover, metformin is believed to activate the AMPK-dependent pathways in the neural stem cells and to decrease BACE1 expression, thus leading to a reduction of Aβ levels. Additionally, metformin decreases the activity of acetylcholinesterase (AChE), which plays a crucial role in the degradation of acetylcholine [129]. Acetylcholine, in turn, is a neurotransmitter that is associated with learning and memory. Although metformin’s properties sound very auspicious and promising, its mechanisms of action in neurodegenerative diseases are not fully understood yet. In animal models, metformin’s effects on cognitive decline are inconsistent. It seems that many factors, such as sex, diet or obesity may have an influence on its outcome. In human studies, in turn, metformin mostly showed a positive influence [130]. Additionally, it is still not clear what dose of the drug should be provided to an individual to obtain its positive results. AChE inhibitors other than metformin are in use to get further from demise caused by AD and ALD [131,132].

## 7. Conclusions

Up to date, there is no causative therapy for Alzheimer’s disease. Surely, there are drugs widely used among those individuals who suffer from the disease, e.g., rivastigmine, memantine, donepezil, and galantamine [131], but they are not sufficient and cause many side effects, such as dizziness, loss of appetite, insomnia, diarrhea, vomiting, or even bradycardia, and syncopes. Moreover, since these drugs do not target the direct causes of AD, they provide symptomatic treatment only. Additionally, they have a tendency to limit their therapeutic effectiveness over time [132]. Undoubtedly, scientists have been putting enormous effort into finding effective prevention, therapy, and understanding Alzheimer’s disease better. However, the index of success is still unsatisfying and there are still too many unknowns vs. knowns about AD regarding not only humans but also (or rather: especially) our best friends and life companions, animals. Nowadays, they are integral members of human households, therefore a better understanding of how they age should be vital for us in order to improve their lives’ comfort. The use of advanced in vivo models and extensive pre-clinical studies needs to be accomplished to increase the likelihood of success in a clinical setup.

## Figures and Tables

**Table 1:** Summary and comparison of Alzheimer’s and Alzheimer’s-like diseases symptoms.

Disease	Species	Symptoms		Literature
		Neuropathological	Behavioral	
**Alzheimer’s disease**	Human	Oxidative stress Neuroinflammation Disruption of neuronal cytoskeleton Impaired neuronal transport Synaptic dysfunction Senile plaques and Aβ aggregates NFTs, hyperphosphorylated tau accumulations Brain atrophy Neuronal loss	Loss of cognition and recognition Disorientation Apathy Poor judgment Intensified aggression or passiveness Changes in interest in food Poor judgment Difficulties in speaking, swallowing and walking Worsening memory	[18,19,21,22,23,24,25,56]
**Alzheimer’s-like disease**	Domestic cat	Aβ oligomers aggregates in cats aged 8 and more Senile plaques in cats aged 10 and more NFTs only in the presence of Aβ Neuronal loss Neuronal degradation	Spatial disorientation or confusion Altered social relationships Intensified aggression or passiveness Changes in daily schedule and wake-sleep pattern Changes in interest in food Decreased grooming Inappropriate vocalization	[66,67,68,69,70,71]
	Camel	Diffuse-type senile plaques in the cerebral cortex	**No data**	[91]
	Elephant	Aβ cerebral deposition		[90]
	Cattle	Aβ cerebral deposition		[89]
	Horse	Tau accumulations Axonal transport deficiencies Diffuse Aβ plaques		[87]
	Sea lion	CSF markers which are found in AD		[92]
	Dolphin	Amyloid and tau pathology in the brain		[92]
	Gorilla	Diffuse-type senile plaques Vascular amyloid		[95,97]
	Baboon	Diffuse-type senile plaques Vascular amyloid Hyperphosphorylated tau in the oldest individuals		[93,98]
	Squirrel monkey	Senile plaques Diffuse-type senile plaques Vascular amyloid		[94]
	Chimpanzee	Diffuse-type senile plaques Vascular amyloid		[96]
	Macaque	Senile plaques Vascular amyloid		[95]
	Sheep	NFTs Aβ cerebral deposition		[88]
	Cheetah	Amyloid plaques NFTs		[20]
	Bears	Amyloid plaques NFTs		[20]
	Goats	NFTs		[20]
**Canine Cognitive Dysfunction (CCD)**	Dog	Cortical atrophy Dysfunction in the neurotransmitter systems Increased oxidative damage Extracellular deposition of diffuse Aβ Neuronal loss Decreased neurogenesis Tau abnormalities, but not NFTs Ventricular enlargement Oligomers of Aβ in the CSF	Loss of cognition and recognition Loss of house training Disorientation Changes in their sleep-wake cycle	[74,75,76,77,78,79,80,81,82,84,85,86]

## Abbreviations

AChE	Acetylcholinesterase
AD	Alzheimer’s disease
ADAM	A disintegrin- and metalloproteinase-family enzymes
AICD	APP intracellular domain
ALD	Alzheimer’s–like disease
APP	Amyloid precursor protein
Aβ	Amyloid β
ROS	Reactive oxygen species
BACE1	Beta-secretase 1
BBB	Blood brain barrier
C83	83-amino acid C-terminal fragment
C99	99-amino acid C terminal fragment
CNS	Central nervous system
CSF	Cerebrospinal fluid
CTF83	83-amino acid C-terminal fragment
FDG-PET	Fluorodeoxyglucose positron emission tomography
GSK-3β	Glycogen synthase kinase-3 beta
HCSMA	Hereditary canine spinal muscular atrophy
MAP	Microtubule associated protein
MCI	Mild cognitive impairment
MHC	Major histocompatibility complex
MND	Motor neuron disease
MRI	Magnetic resonance imaging
NFT	Neurofibrillary tangles
PET	Positron emission tomography
RNS	Reactive nitrogen species
sAPPα	Soluble amyloid precursor protein α
sAPPβ	Soluble amyloid precursor protein β
SP	Senile plaques
SR	Scavenger receptors
T2D	Type 2 diabetes mellitus
T2DM	Type 2 diabetes mellitus
TLR	Toll-like receptors
TPK	Tau protein kinase
